# Healthcare Facilities Established by the Eastern Orthodox Church in Sub-Saharan Africa: A Questionnaire Survey

**DOI:** 10.7759/cureus.85264

**Published:** 2025-06-02

**Authors:** Lykourgos Christos Alexakis, George Binna Sande, Angeliki Konstantinou, Dionysia Filaditaki, Dominique Sangwa Ngoie, Ioannis Apostolakis, Aristomenis I Syngelakis

**Affiliations:** 1 School of Social Sciences, Hellenic Open University, Patras, GRC; 2 Family Medicine, Uganda Orthodox Medical Bureau, Kampala, UGA; 3 Anesthesiology, Sankt Katharinen Hospital, Frankfurt, DEU; 4 Psychiatry, Lausanne University Hospital, Lausanne, CHE; 5 Family Medicine, Hôpital Orthodoxe De Kolwezi St Cosmas Et Damien, Kolwezi, COD; 6 School of Medicine, National and Kapodistrian University of Athens, Athens, GRC; 7 School of Dentistry, National and Kapodistrian University of Athens, Athens, GRC; 8 School of Dentistry, European University Cyprus, Engomi, CYP

**Keywords:** africa, africa south of the sahara, eastern orthodox church, faith-based organizations, health care facilities manpower and services, health facilities, health personnel, health services, religious missions

## Abstract

Introduction: The Orthodox Church has a long presence in the African continent, and its philanthropic activity includes, among others, schools, universities, orphanages, and health centers. Although there are different healthcare facilities established by the Orthodox Church in Sub-Saharan Africa, there is a lack of detailed information concerning their current status. The study objective was to clarify (a) the geographical location of these facilities, (b) their operational status, (c) their existing infrastructure, (d) their staffing, and (e) the services provided.

Methods: A questionnaire based on the World Health Organisation's Service Availability and Readiness Assessment tool (SARA) was sent to the respective archdioceses and dioceses of the Eastern Orthodox Church in Sub-Saharan Africa, which are under the jurisdiction of the Patriarchate of Alexandria and All Africa.

Results: The response rate was 90% (27 out of the 30 archdioceses and dioceses). In total, 52 healthcare facilities were registered (two hospitals, 40 health centres, and 10 health posts/clinics). In Uganda, 17 healthcare facilities were confirmed, in Kenya 10, in Madagascar six, in Tanzania four, in Cameroon four, in the Democratic Republic of Congo three, in Sierra Leone three, in Zambia two, in Zimbabwe one, in Malawi one, and in Congo-Brazaville one. Out of the 52 facilities, 18 were urban while 34 were rural. From the 52 facilities identified, 34 were operational despite the COVID-19 pandemic. Out of these 34 operational facilities, 23 provided only outpatient clinics, while in 11, hospitalisation/inpatient monitoring was also possible. Various healthcare services were provided, mainly at the primary care level. Secondary level of care, such as surgical and obstetric services, was also provided in the larger facilities. Financing was mainly from donations, fees paid by the patients, and in a few cases, government contributions.

Conclusion: The Orthodox Church’s philanthropic activity in the field of healthcare in Sub-Saharan Africa is extensive. The 52 healthcare facilities established in 11 countries constitute a decentralised network covering a large part of Sub-Saharan Africa. This network might be a useful collaborating partner for universities and research institutions developing projects in the fields of tropical medicine, infectious diseases, remote medicine, or climate change and health.

## Introduction

The Eastern Orthodox Church (officially, the Orthodox Catholic Church), also known as the Orthodox Church or the Greek Orthodox Church, is one of the Christian churches with missionary activities in Sub-Saharan Africa. Many parishes have been established since the mid-20th century, and they fall under the jurisdiction of the Patriarchate of Alexandria and All Africa [1]. The Patriarchate of Alexandria is the second in rank of the 14 autocephalous Orthodox Churches, which in their totality constitute Orthodoxy, one of the three essential doctrines of Christianity, the other two being Roman Catholicism and Protestantism [2].

Healthcare services provision for patients in need, without religious or other discrimination, is often part of the charity work performed by the Orthodox Church in Africa [3-5]. However, the available information is often incomplete, fragmented, or outdated, and there seems to be a lack of information about their current operational status, their available infrastructure, and the services they provide.

In order to review the situation and to assess the novelty of a study in this field, a literature review was performed in PubMed on October 18, 2022, with the search terms: “Orthodox Church” which gave 215 results, “Orthodox Church AND Africa” which gave 27 results, as well as “Orthodox Church AND Health Care Facilities” which gave 17 results. Additionally, the following Medical Subject Heading (MeSH) terms using Boolean commands were checked: “Eastern Orthodoxy” which gave 130 results, and “Eastern Orthodoxy AND Africa” which gave five results. All articles were reviewed, and no publication was identified among them dealing with subjects such as a systematic registration, mapping, or assessment of the Orthodox Church healthcare facilities in Africa. The only relevant article was a letter to the editor analysing the diagnoses of 437 patients examined by a volunteer medical team from Greece, during a 30-day-long mission in an Orthodox Church health center located in Kinshasa, Democratic Republic of Congo [6].

From the literature review results, it seems that the healthcare service provided by the Orthodox Church in Africa is an understudied field despite the long presence of the Orthodox Church on the continent, the vast geographic area involved, and the huge population of potential beneficiaries. This study was an initial attempt to bridge this research gap.

## Materials and methods

Study design

The research question/ study objective was to clarify: (a) the geographical location of the healthcare facilities established by the Orthodox Church in Sub-Saharan Africa, (b) their current operational status, (c) their available equipment/infrastructure, (d) their staffing, and (e) the services they provide.

The study included a descriptive questionnaire survey collecting basic data on healthcare facilities and their infrastructure and services. It did not include any individual patient data collection or any intervention on human subjects, and hence, no ethical committee approval was required. The study protocol was approved by the School of Social Sciences, Hellenic Open University, in October 2022. Approval for the study was obtained by His Beatitude Theodoros II, Pope and Patriarch of Alexandria and All Africa of the Orthodox Church. Subsequently, the head of each archdiocese/metropolis and each diocese included in the study sample was informed about the study, and they provided consent before filling out the questionnaire.

Eligibility criteria and sample size

All the archdioceses/metropolises and dioceses of the Eastern Orthodox Church having spiritual jurisdiction in at least a country or other geographical area (e.g., city, region, county) of Sub-Saharan Africa were included in the study. Newly established dioceses without an appointed bishop were excluded. For the sample determination, convenience sampling was used. The study sample included all 21 archdioceses/metropolises and nine dioceses, which were identified from the website of the Patriarchate of Alexandria and All Africa as fulfilling the above inclusion and exclusion criteria (Table 1) [7]. Our definition of Sub-Saharan Africa included all countries of the African continent, except Egypt, Libya, Tunisia, Algeria, Morocco, Western Sahara, and the island of Saint Helena, a British overseas territory.

**Table 1:** Archdioceses and dioceses included in the study * only Mauritania is included

Archdiocese/Metropolis or Diocese	Spiritual jurisdiction
Archdiocese of Nairobi	Kenya
Archdiocese of Zimbabwe and Angola	Zimbabwe and Angola
Archdiocese of Nigeria	Nigeria, Niger, Benin, Togo
Archdiocese of Cape of Good Hope	Namibia, Swaziland, Lesoto, areas of West and East Cape, Port Elizabeth, Natal, East London, Bloemfontein, Welkom, George, Knysna, Kimberley, Natal, Pietermaritzburg
Archdiocese of Kampala	Uganda
Archdiocese of Guinea	Sierra Leone, Liberia, Guinea, Guinea-Bissau, Gambia, Senegal, Cape Verde
Archdiocese of Irinopolis	Tanzania, Seychelles
Archdiocese of Johannesburg and Pretoria	Greater areas of Johannesburg and Pretoria, Mpumalanga, and Limpopo provinces
Archdiocese of Antananarivo and Northern Madagascar	Madagascar, Mauritius, Reunion, Comores, Mayotte
Archdiocese of Cameroon	Cameroon, Chad, Central African Republic, Equatorial Guinea, São Tomé and Príncipe
Metropolis of Katanga	Katanga, Democratic Republic of Congo
Archdiocese of Botswana	Botswana, Free State of South Africa
Archdiocese of Nubia	Sudan, South Sudan
Archdiocese of Zambia	Zambia
Archdiocese of Burundi and Rwanda	Burundi, Rwanda
Archdiocese of Aksum	Ethiopia, Eritrea, Djibuti, Somalia
Archdiocese of Kinshasa	Kinshasa, Democratic Republic of Congo
Archdiocese of Accra	Ghana, Ivory Coast, Mali, Burkina Faso
Metropolis of Kananga	Kananga, Democratic Republic of Congo
Archdiocese of Carthage*	Tunisia, Morocco, Algeria, Mauritania*
Archdiocese of Congo-Brazzaville and Gabon	Republic of the Congo (Brazzaville), Gabon
Diocese of Nieri and Mount Kenya	Nieri and Mount Kenya
Diocese of Arusha and Central Tanzania	Arusha and Central Tanzania
Diocese of Jinja and Eastern Uganda	Jinja and East Uganda
Diocese of Malawi	Malawi
Diocese of Toliara and Southern Madagascar	Toliara and Southern Madagascar
Diocese of Kisumu and Western Kenya	Kisumu and Western Kenya
Diocese of Gulu and Northern Uganda	Gulu and Northern Uganda
Diocese of Bukoba and Western Tanzania	Bukoba and Western Tanzania
Diocese of Mozambique	Mozambique

Study tool

The questionnaire was created based on the World Health Organisation’s Service Availability and Readiness Assessment tool (SARA) [8]. Only some sections from the SARA core instrument were included, which were selected as per study objectives. The questions selected were adapted in order to create a self-administered questionnaire. Some additional exploratory questions were also added at the end.

English and French language versions of the questionnaire (See Appendices), along with an accompanying introductory letter explaining the study addressed to the Archbishop or Bishop, were sent as hard copies by post as well as digitally via email to the heads or (in few cases) their designated delegates of all archdioceses/metropolises and dioceses included in the study. Additionally, email and telephone follow-up were arranged. Questionnaire distribution and data collection took place from December 2022 to June 2023. No further data were submitted to the researchers during the three-month follow-up period from July to September 2023.

Bias

The constructed questionnaire was reviewed for face validity (relevance, format, readability, clarity, appropriateness for the intended audience) and content validity (relevance of questions, coverage of the aspects studied, representativeness of the construct being measured) by two of the authors (LCA, AK). Pilot testing included sending the questionnaire initially only to the archdioceses/metropolises and dioceses of the sample, which were located in four countries (Madagascar, Tanzania, Kenya, Democratic Republic of Congo). During this step, completed questionnaires were received from five different archdioceses/metropolises and dioceses located in these countries. The responses and captured data were compared for consistency. No changes were required since the consistency of answers was confirmed. Subsequently, the same questionnaire was sent to the rest of the study sample, and the responses obtained were included together with the pilot study responses in the study analysis. 

Apart from the information in the answered questionnaires, data were also collected in the form of free text from email answers and activity report letters received by the archdioceses/metropolises and dioceses. Additionally, some data were collected over the telephone via unstructured interviews with Archbishops, Bishops, or appropriate representatives.

Statistical methods

All data were entered in a study-specific spreadsheet and were analysed with descriptive statistical methods. Depending on the variables, measures of frequency distribution, central tendency, and variability were calculated, which included count, mean, median, mode, minimum, and maximum values as well as standard deviation (SD). The software used was Numbers, version 12.2.1 (7035.0.161) (Apple Inc., Cupertino, California, United States).

## Results

The response rate to the survey was 90% (27 of the 30 archdioceses/metropolises and dioceses). From the 30 archdioceses/metropolises and dioceses, 18 confirmed they owned healthcare facilities, nine confirmed there were none in their area of jurisdiction, while three did not provide a response. A list of healthcare facilities in the archdioceses and dioceses that responded to the questionnaire is given in Table 2.

**Table 2:** Healthcare facilities per archdiocese and diocese

Archdiocese/Metropolis or Diocese	Number and type of healthcare facilities (Location)
Archdiocese of Nairobi	9 Health Centers (Kamangu ,Thogoto, Kanyanjara, Nderi, Riruta, Ngecha, Kimende, Kibera, Ongata Rongai)
Archdiocese of Zimbabwe and Angola	1 Health Center (Harare)
Archdiocese of Nigeria	UNKNOWN/INSUFFICIENT DATA
Archdiocese of Cape of Good Hope	NONE
Archdiocese of Kampala	1 Hospital (Kampala), 8 Health Centers (Bulami, Degeya, Mpigi, Ngombe, Monde, Kireku, Bukuya, Magoma)
Archdiocese of Guinea	3 Health Posts in Sierra Leone (2 in Freetown, 1 in Waterloo)
Archdiocese of Irinopolis	2 Health Posts (Morogoro, Kilosa)
Archdiocese of Johannesburg and Pretoria	NONE
Archdiocese of Antananarivo and Northern Madagascar	3 Health Centers (Alasora in Antananarivo, Zoma Bealoka, Soavinarivo)
Archdiocese of Cameroon	4 Health Posts (Nkolgok, Katrang, Ndoukoula, Guere)
Metropolis of Katanga	1 Hospital (Kolwezi)
Archdiocese of Botswana	NONE
Archdiocese of Nubia	NONE
Archdiocese of Zambia	2 Health Centers (Lusaka, Chirundu)
Archdiocese of Burundi and Rwanda	UNKNOWN/INSUFFICIENT DATA
Archdiocese of Aksum	NONE
Archdiocese of Kinshasa	1 Health Center (Mont Ngafula in Kinshasa), 1 Health Post (Shamana)
Archdiocese of Accra	Available spaces in schools and churches are used for mobile clinics occasionally
Metropolis of Kananga	NONE
Archdiocese of Carthage	NONE
Archdiocese of Congo-Brazzaville and Gabon	1 Health Center (Pointe-Noire)
Diocese of Nieri and Mount Kenya	UNKNOWN/INSUFFICIENT DATA
Diocese of Arusha and Central Tanzania	1 Health Center (Kidamali)
Diocese of Jinja and Eastern Uganda	6 Health Centers (Abuchete, Bugolo, Katente, Nsinze, Lwaniha, Nawango)
Diocese of Malawi	1 Health Post (Kabodzi)
Diocese of Toliara and Southern Madagascar	1 Health Center (Toliara), 2 Health Posts (Beravy, Abaskibo/Ambasikibo)
Diocese of Kisumu and Western Kenya	1 Health Center (Chavogere)
Diocese of Gulu and Northern Uganda	2 Health Centers (Gulu, Akonyibedo)
Diocese of Bukoba and Western Tanzania	1 Health Center (Bukoba)
Diocese of Mozambique	NONE

Descriptive data

In total, 52 healthcare facilities belonging to the Eastern Orthodox Church were identified in Sub-Saharan Africa. Of these, two (3.9%) were hospitals, 40 (76.9%) were health centres, and 10 (19.2%) were health posts. Out of the 52 facilities, 34 (65.4%) were operating at the time of the study, while 18 (34.6%) were not. Of these 18 non-functioning facilities, three were newly constructed and were expected to start providing services within the next few months, two were under construction, and 13 were closed for various reasons.

**Figure 1:**
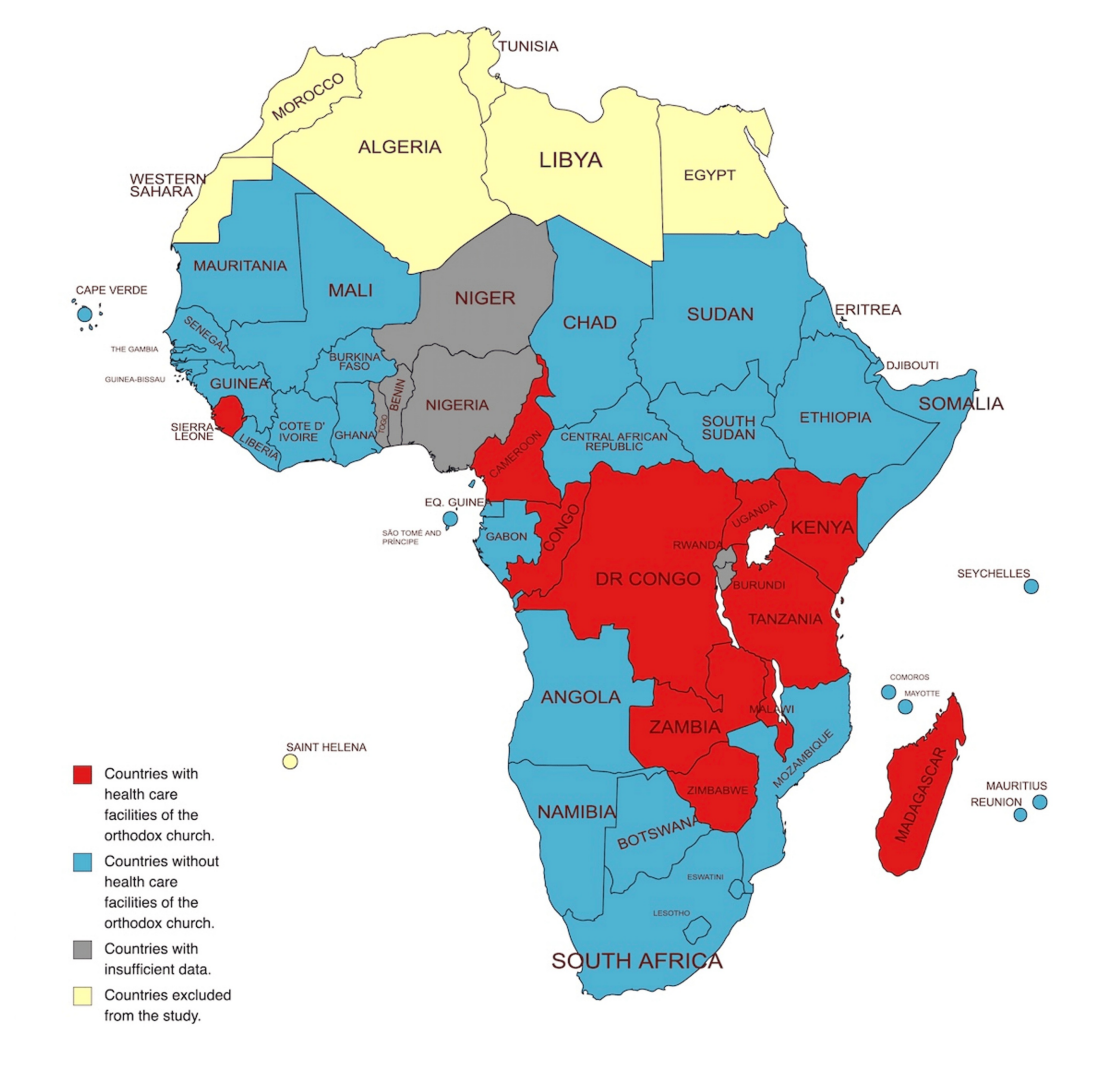
Presence of heathcare facilities of the Eastern Orthodox Church in countries of Sub-Saharan Africa Image Credit: Lykourgos Christos Alexakis, created with mapchart.net and edited with Apple Preview (Apple Inc., Cupertino, California, United States)

The geographical distribution of the 52 healthcare facilities is given in Table 3 and Figure 1. Uganda had the highest number of facilities (n=17, 32.7%).

**Table 3:** Healthcare facilities run by the Eastern Orthodox Church in Sub-Saharan African distributed according to country ^*^New health care facilities which were expected to be operational soon; ^†^Closed healthcare facilities that were not operating at the time of the study; ^‡^Healthcare facilities under construction

Country	Health Posts, number (location)	Health Centers, number (location)	Hospitals, number (location)	Other
Madagascar	2 (Beravy*, Abaskibo/Ambasikibo*)	4 (Antananarivo, Toliara, Soavinarivo, Zoma Bealoka)	-	-
Tanzania	2 (Morogoro†, Kilosa^†^)	2 (Kidamali, Bukoba)	-	-
Democratic Republic of Congo	1 (Shamana^†^)	1 (Kinshasa*)	1 (Kolwezi)	-
Kenya	-	10 (Chavogere, Kamangu, Thogoto, Kanyanjara, Nderi, Riruta, Ngecha, Kimende, Kibera, Ongata Rongai^†^)	-	-
Zimbabwe	-	1 (Harare)	-	-
Cameroon	4 (Nkolgok, Katrang, Ndoukoula^†^, Guere^†^)	-	-	-
Malawi	1 (Kabodzi^†^)	-	-	-
Uganda	-	16 (Akonyibedo, Bulami, Degeya, Mpigi, Ngombe, Monde, Kireku, Bukuya^†^, Magoma†, Abuchete, Bugolo, Katente, Nsinze, Lwaniha^†^, Nawango^†^, Gulu^†^)	1 (Kampala)	-
Zambia	-	2 (Lusaka^†^,Chirundu^‡^)	-	-
Sierra Leone	-	3 (two in Freetown, one in Waterloo)	-	-
Republic of the Congo/Brazzaville	-	1 (Pointe-Noire^‡^)	-	-
Ghana, Mali, Ivory Coast, Burkina Faso	-	-	-	Schools and churches of Accra Archdiocese were available for mobile clinics.

**Figure 2:**
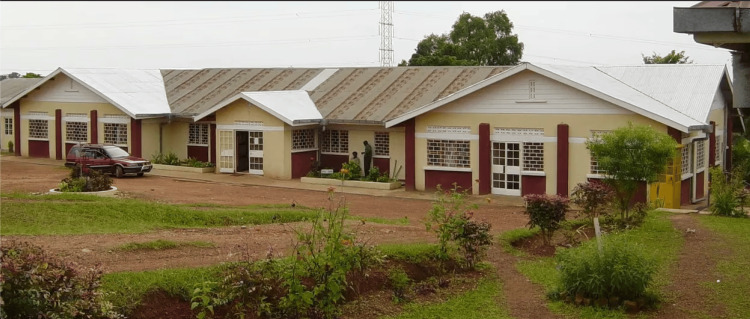
The hospital in Kampala, Uganda Image Credit: Center for International Solidarity and Cooperation "Saints Kosmas & Damianos”/KEDAS, Greece; published with permission.

**Figure 3:**
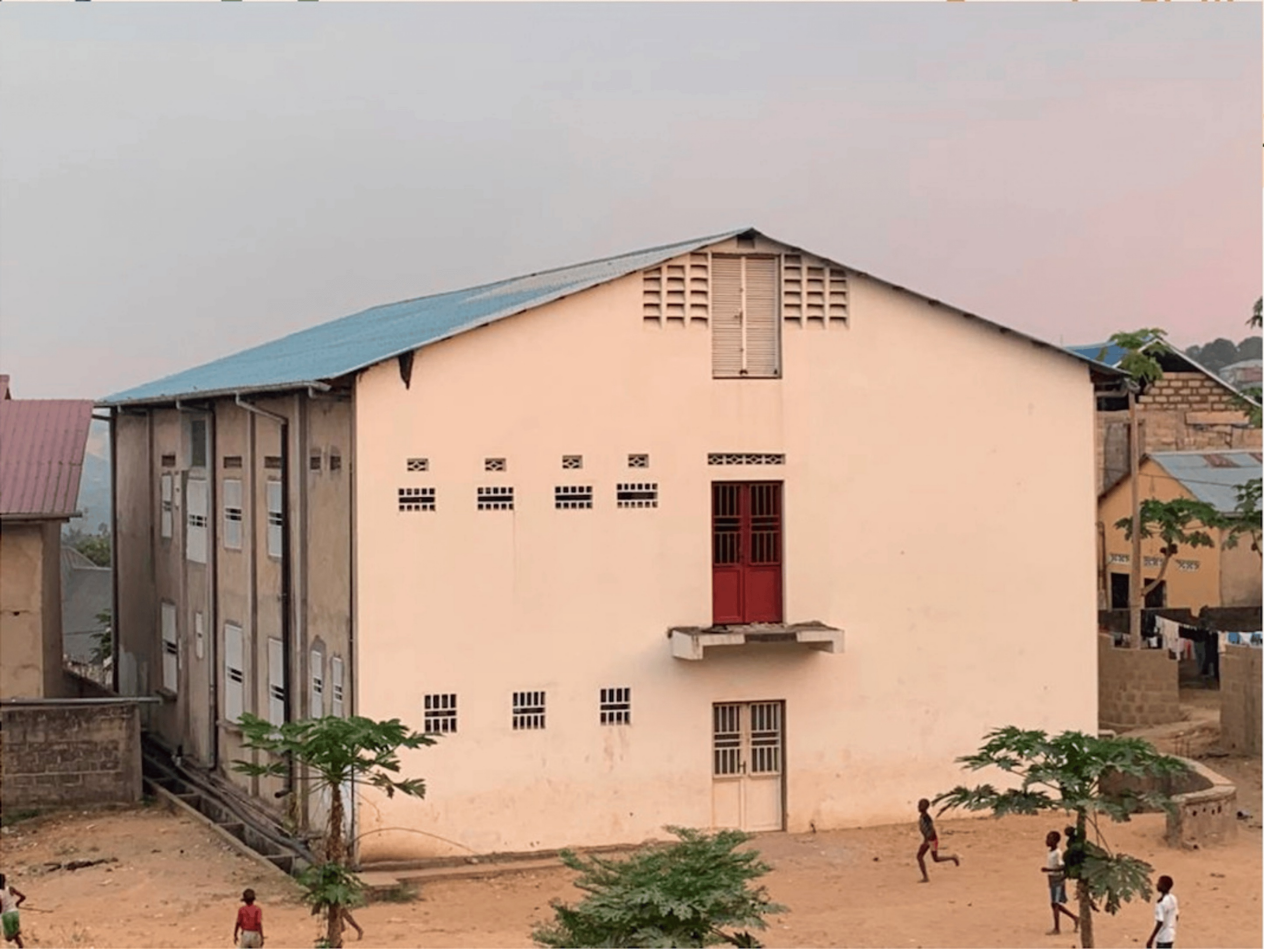
The health center at Mont Ngafula in Kinshasa, Democratic Republic of Congo, that was non-functioning at the time of the study Image Credit: Orthodox Archdiocese of Kinshasa; published with permission.

Regarding the location, 18 (34.6%) units were urban and 34 (65.4%) were rural. Of the 34 healthcare facilities that were operating, 23 (67.6%) provided exclusively outpatient services, while in 11 (32.4%), hospital beds were available and inpatient monitoring was possible. The 34 facilities that were operating at the time of the study had 224 hospital beds in total (including inpatient beds and short monitoring/observational care beds), with a mean of 6.8 beds each (minimum: 0, maximum: 100, median: 2, mode: 0, SD: 18.24). As expected, the two hospitals had the highest number of beds. The hospital in Kolwezi, Democratic Republic of Congo, had 100 beds, and the hospital in Kampala, Uganda (Figure 2) had 36 beds. Of the 18 closed health facilities, only the health center in Lwaniha, Uganda, had three beds, and the health center in Kinshasa, Democratic Republic of Congo (Figure 3) had nine beds. 

For 33 out of the 34 operating facilities, detailed data on personnel were available. Nursing professionals were the most common personnel category, followed by generalist medical doctors, laboratory technicians, non-physician clinicians/paramedical professionals, midwifery professionals, specialist medical doctors, pharmacists, and community health workers (Table 4).

**Table 4:** Distribution of the staff according to category in the 33 healthcare facilities with available personnel data

Staff Category	Number of Staff	Mean	Minimum	Maximum	Median	Mode	Standard Deviation
Generalist (non specialist) medical doctors	32	0.97	0	7	1	0	1.61
Specialist medical doctors	17	0.52	0	6	0	0	1.37
Non-physician clinicians/paramedical professionals	28	0.85	0	10	0	0	1.97
Nursing professionals	88	2.67	1	22	1	1	3.93
Midwifery professionals	24	0.73	0	7	0	0	1.53
Pharmacists	11	0.34	0	2	0	0	0.54
Laboratory technicians	29	0.88	0	5	1	1	1.14
Community health workers	11	0.34	0	3	0	0	0.78

In 22 of the facilities that were operating, the number of patient consultations that took place the previous month was recorded. The total for all 22 healthcare units was 9543 consultations during one month of operation, although the actual month counted varied per facility (mean: 434, minimum: 10, maximum: 2627, median: 150, mode: 0, SD: 688).

Regarding patient amenities, the following was found. Out of the 34 operational facilities, one (2.9%) was open for five to eight hours a day on average, 10 (29.4%) were open for 9-16 hours a day, 13 (38.2%) were open 24 hours daily, while for the remaining 10 (29.4%) facilities, no data were available. No healthcare facilities were open for 17-23 hours daily, nor for four hours or less daily. Out of the 34 facilities, in one (2.9%), the examination room did not provide any protection of the patient's privacy; in one (2.9%), it provided only visual protection; in 22 (64.7%), the examination room provided both visual and acoustic protection of the patient’s privacy; while for the remaining 10 (29.4%) facilities, this information was not available.

Concerning the most commonly used water source at the time, out of the 34 operating healthcare facilities, in eight (23.5%), water was piped into the facility, in one (2.9%) water was piped into facility and rainwater collection was used; in one (2.9%), water was piped into facility and tubewell/borehole was used; in four (11.8%) facilities, the water source was a tubewell/borehole; in one (2.9%) facility, only rainwater collection was used; in two (5.9%), a tubewell/borehole along with rainwater collection; in six (17.6%) facilities, a protected dug well and rainwater collection was used as a water source; in one (2.9%) facility, they used tubewell/borehole, protected dug well, and rainwater collection. In the remaining 10 (29.4%) facilities, this information was not available. In the 18 closed facilities, the most commonly used water source for one (5.6%) was water piped into the facility and rainwater collection; for five (27.8%), there was a protected dug well along with rainwater collection. For the remaining 12 (66.7%) closed facilities, this information was not available.

The available type of toilet for use by outpatients in the 34 operating facilities was flush toilet in four (11.8%) facilities, ventilated improved pit latrine (VIPL) in 13 (38.2%), flush toilet and VIPL in three (8.8%), pit latrine with slab in three (8.8%), open pit latrine in one (2.9%), while for 10 (29.4%) facilities this information was not available. Of the 18 closed facilities, one (5.6%) had a flush toilet for outpatient use, five (27.8%) had VIPL, and for 12 (66.7%), this information was not available.

The power supply infrastructure, medicine storage, ambulance availability, and communication equipment of the 34 operational facilities, according to the collected questionnaire responses, are provided in Table 5.

**Table 5:** Infrastructure available in the 34 operating healthcare facilities

Infrastructure	Number of facilities where this is available (YES)	Number of facilities where this is not available (NO)	Number of facilities where the availability is not known (UNKNOWN)
Functioning land line telephone	8	16	10
Functioning cellular telephone	18	6	10
Functioning short-wave radio for radio calls	1	23	10
Functioning computer	12	12	10
Functional ambulance or other vehicle for emergency transportation for patients stationed at facility	5	19	10
Access to an ambulance or other vehicle for emergency transport for patients at another facility in near proximity	9	15	10
Electricity from any source (e.g. electricity grid, generator, solar, other)	24 (grid: 21, solar: 3)	0	10
Secondary or backup source of electricity	12 (generator: 8, solar: 4, rechargeable lantern: 1)	11	10
Medicine stock	26	0	8
Refrigerator/cold storage for medicines or vaccines	10	13	11

Many different services were provided by the 34 operational healthcare facilities. Although the majority of facilities provided primary medical care, some larger facilities also provided secondary level of care, such as surgical services (minor surgery or cesarian section), obstetric services (delivery or newborn care), and transfusion services (Table 6).

**Table 6:** Services provided by the 34 operational healthcare facilities

Services Provided	Number of facilities where this is available (YES)	Number of facilities where this is not available (NO)	Number of facilities where the availability is not known (UNKNOWN)
Family planning services.	15	9	10
Antenatal care services	21	2	11
Prevention of mother-to-child transmission of HIV	9	15	10
Delivery (including normal delivery, basic emergency obstetric care, and/or comprehensive emergency obstetric care) and/or newborn care services	7	16	11
Immunization services	14	10	10
Preventative and curative care services for children under five years of age	23	0	11
Adolescent health services	9	15	10
HIV counseling and testing services	13	9	12
HIV/AIDS antiretroviral prescription or treatment follow-up	5	17	12
Diagnosis or treatment of sexually transmitted infections other than HIV	18	2	14
Diagnosis, treatment prescription, or treatment follow-up of tuberculosis	9	15	10
Diagnosis or treatment of malaria	24	0	10
Diagnosis or management of non-communicable diseases, such as diabetes, cardiovascular disease, chronic respiratory disease	11	13	10
Surgical services (including minor surgery such as suturing, circumcision, wound debridement, etc.), or caesarean section	13	11	10
A staff member trained in surgery, including caesarean section (clinical officer, general physician, or surgeon)	12	12	10
A staff member trained in anaesthesia (nurse, clinical officer, general physician, surgeon, or anaesthesiologist)	7	17	10
Blood transfusion services	3	21	10
Diagnostic testing (including any rapid diagnostic testing)	22	3	9
Ziehl-Neelsen testing for Tuberculosis (AFB) onsite or offsite	9	15	10
Diagnostic X-ray	4	23	7
Diagnostic ultrasound	8	17	9
Diagnostic computerized tomography (CT scan)	0	27	7
Diagnostic electrocardiogram (ECG)	5	18	11

Of the 34 operational facilities, in 24 (70.6%), patients paid a monetary contribution for the services provided; in six (17.6%), they did not pay any contribution for the services; and no information was available for four (11.8%) facilities. Concerning medicine prescriptions, in eight, (23.5%) facilities, prescribed medicine were provided free of charge by the facility; in 14 (41.2%), patients bought them from the facility at low/reduced prices; in two (5.9%), patients bought them from the facility at low/reduced prices and when the drugs were not available there, they bought them from local pharmacies; in two (5.9%), patients bought them from the facility at normal prices; in four (11.8%), patients bought them from the facility at normal prices and when the drugs were not available there, they bought them from local pharmacies; for four (11.8%) facilities, this information was not available. No facility reported patients buying the prescribed medicines exclusively from the local market/pharmacies.

The funding sources of the 34 operational healthcare facilities were diverse. In 11 (32.4%) facilities, financing was from donations; in nine (26.5%) facilities, it was from donations and the financial participation of the patients; in one (2.9%), funding came from the diocese and the income from the operation of the hospital; in one (2.9%) facility, funding came from the diocese and donations; in four (11.8%) facilities, funding came from donations, private payments, and some kind of state aid; in six (17.6%) facilities, funding came from patient payments (for consultations, laboratory tests, hospitalisation, purchased medicines); in one (2.9%) facility, funding came from the archdiocese and patient payments (for consultation and purchased medicines); for one (2.9%) facility, this information was not available.

## Discussion

A mapping of the healthcare facilities available in a given geographic area is a useful tool for healthcare services planning, especially in Africa, where access to the public health system is not always evident [9]. Our study shows that the healthcare facilities established by the Orthodox Church in Sub-Saharan Africa are numerous. In total, 52 facilities were registered, which are distributed in 11 countries. This number is impressive considering the fact that the primary activity of the church is missionary and healthcare is only a part of the charity work carried out, which also includes orphanages, nursing homes, schools, vocational schools, and even universities [10,11].

**Figure 4:**
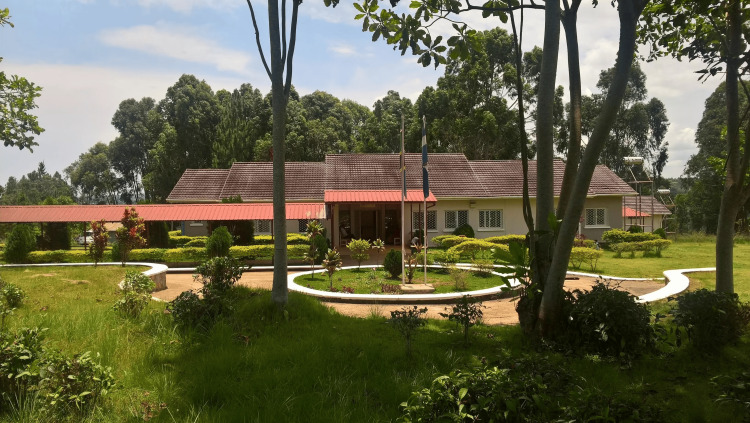
The health center in Bukoba, Tanzania Image Credit: Center for International Solidarity and  Cooperation "Saints Kosmas & Damianos”/KEDAS, Greece; published with permission.

Establishing and operating a healthcare facility is challenging. In addition to the initial construction and equipment cost, maintenance should be taken into account. Staff salaries are an inelastic expense that exists even when patient attendance is reduced. This constitutes the largest part of the fixed costs of a facility [12]. For this reason, regular periodic financial support from orthodox parishes abroad, small NGOs, and missionary organisations, who collect donations, is often necessary for the continuous provision of these healthcare services [13-15]. An example of this model of financing is the health center in Bukoba, Tanzania (Figure 4). Apart from funding from the diocese of Bukoba, the health center is also financially supported by orthodox parishes in the United States (St Paul, Irvine and St Barbara, Santa Barbara, California) as well as the Center for International Solidarity and Cooperation "Saints Kosmas & Damianos" (KEDAS), which is an NGO based in Greece [13]. Occasionally, individual donors contact a diocese or archdiocese directly through their official website, social media pages, or through the website of the Patriarchate of Alexandria and All Africa [4,7,16-17].

**Figure 5:**
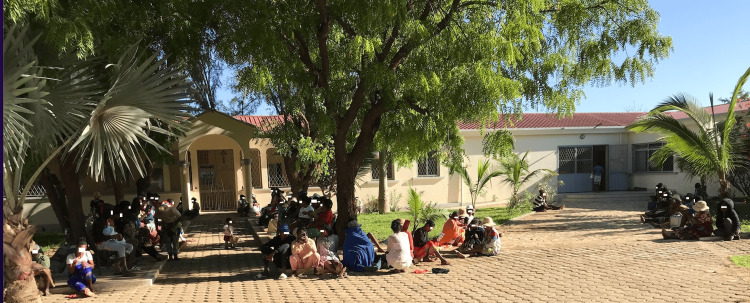
The health center in Toliara, Southern Madagascar Image Credit: International Humanitarian Aid “Mother of God”, Cyprus; published with permission.

Despite the limited funding, the medical care provided through the Orthodox Church facilities is seen to be cost-effective. A typical example is the health center in Toliara, Madagascar (Figure 5). The center’s monthly expense of €5000 is covered regularly by the NGO International Humanitarian Aid “Mother of God”, Cyprus [14]. This amount includes the salaries of the staff (three general doctors, one specialist doctor, six nurses, one pharmacist, one lab technician), utilities (e.g. electricity, fuel), and the cost of medicines, which are dispensed free of charge (personal communication with Bishop of Toliara, Mr Prodromos, May 4, 2023). The health center provides 2000 patient consultations on average per month, so the calculated average cost per consultation, including the cost of all prescribed medications, was €2.5. By comparison, the cost per consultation, including prescribed medications, in a primary healthcare clinic run by students in South Africa in 2017 was £3.54 (ZAR 69.05) [18]. Taking inflation into account, this would be equal to £4.5 in May 2023, corresponding to €5.25.

**Figure 6:**
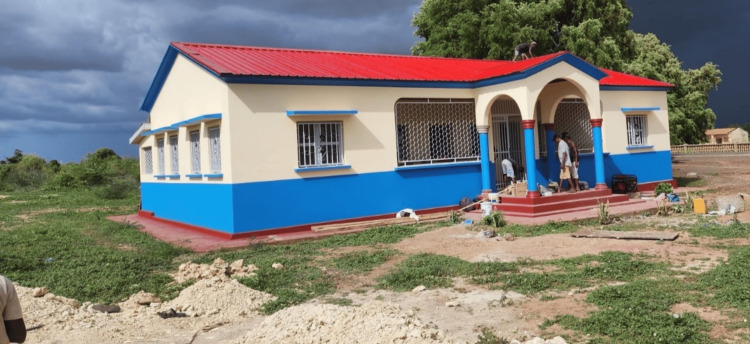
The health post in Abaskibo/Ambasikibo, Madagascar Image Credit: Orthodox Diocese of Toliara and Southern Madagascar; published with permission.

In Madagascar, an issue mentioned was the reluctance of doctors to work in remote healthcare facilities as they could find jobs with better conditions elsewhere. In the facilities located in the northern part of the country belonging to the archdiocese of Antananarivo, employed local doctors did not stay long in their job posts and often resigned. This phenomenon has also been observed in other low-income countries [19]. In southern Madagascar, at the health center of Toliara, in order to retain doctors, a monthly salary of 1,200,000 Ariary (corresponding to €243 Euro in May 2023), which is above the public sector salary, is offered. Also, in the two newly constructed health posts in Beravy and Abaskibo/Ambasikibo (Figure 6), which are remote, an accommodation section for the doctor is included in each facility.

Of particular interest is the healthcare facility coordination model in Uganda. The Uganda Orthodox Medical Bureau, founded as a charitable faith-based NGO, serves as a liaison between the Uganda Orthodox Church, the government of Uganda, donors, and all the healthcare facilities affiliated to the Uganda Orthodox Church [20]. This might be a useful model to adopt either in individual countries of Africa with many Orthodox healthcare facilities or at the regional or even continental level.

The healthcare facilities established by the Orthodox Church in Sub-Saharan Africa constitute a decentralized network covering an extensive geographic area in 11 countries. These healthcare facilities are potentially useful partners for universities and research institutions developing local or international educational and research projects. There are many relevant fields for such collaborations, including, among others, clinical tropical medicine, infectious diseases epidemiology, remote medicine, healthcare in low-resource settings, and climate change-related health issues.

Limitations

This study was a questionnaire survey, and there are many limitations inherent to this research method. More specifically, errors or omissions during the questionnaire design and use, data collection by non-medical personnel, information provided through unstructured text emails, and over the telephone, could have led to measurement errors and bias affecting the quality and completeness of the data. Selection/non-response bias may also be present, as from some Dioceses and Archdioceses, no response was obtained. Additionally, underreporting of data from smaller, closed, or non-operational facilities could have occurred.

## Conclusions

The Orthodox Church’s philanthropic activity in the field of healthcare in Sub-Saharan Africa is extensive. In total, 52 healthcare facilities distributed in 11 countries have been identified. These facilities were located in Uganda, Kenya, Madagascar, Tanzania, Democratic Republic of Congo, Sierra Leone, Zimbabwe, Cameroon, Malawi, Zambia, and the Republic of Congo-Brazzaville. Of the 52 identified, 34 were operating during the period of the study and were providing a variety of primary healthcare services to the local population, at low or zero cost for the patients. The two hospitals in Kampala (Uganda) and in Kolwezi (Democratic Republic of Congo) were also providing a secondary level of care. Funding was, to a great extent, from donations.

This decentralized network of healthcare facilities covering a large part of Sub-Saharan Africa might be a useful collaborating partner for universities and research institutions developing projects in the fields of tropical medicine, infectious diseases, remote medicine, and climate change-related health issues.

## Appendices

All detailed data collected per health care facility, as well as the specific questionnaires used for the study, are available online as supplementary material (https://doi.org/10.6084/m9.figshare.28908485.v2).
